# Short term effects of different omega-3 fatty acid formulation on lipid metabolism in mice fed high or low fat diet

**DOI:** 10.1186/1476-511X-11-70

**Published:** 2012-07-10

**Authors:** Xiao Tang, Zhao-Jie Li, Jie Xu, Yong Xue, Jin-Zhang Li, Jing-Feng Wang, Teruyoshi Yanagita, Chang-Hu Xue, Yu-Ming Wang

**Affiliations:** 1 College of Food Science and Engineering, Ocean University of China, Qingdao, China; 2 Department of Applied Biological Sciences, Saga University, Saga, Japan

**Keywords:** Omega-3 fatty acid, DHA, EPA, Lipid metabolism, Triglycerides, Ethyl ester, Phospholipids

## Abstract

**Background:**

Bioactivities of Docosahexaenoic acid (DHA) and Eicosapentaenoic acid (EPA) depend on their chemical forms. The present study was to investigate short term effects of triglyceride (TG), ethyl ester (EE), free fatty acid (FFA) and phospholipid (PL) forms of omega-3 fatty acid (FA) on lipid metabolism in mice, fed high fat or low fat diet.

**Method:**

Male Balb/c mice were fed with 0.7% different Omega-3 fatty acid formulation: DHA bound free fatty acid (DHA-FFA), DHA bound triglyceride (DHA-TG), DHA bound ethyl ester (DHA-EE) and DHA bound phospholipid (DHA-PL) for 1 week, with dietary fat levels at 5% and 22.5%. Serum and hepatic lipid concentrations were analyzed, as well as the fatty acid composition of liver and brain.

**Result:**

At low fat level, serum total cholesterol (TC) level in mice fed diets with DHA-FFA, DHA-EE and DHA-PL were significantly lower than that in the control group (*P* < 0.05). Hepatic TG level decreased significantly in mice fed diets with DHA-TG (*P <* 0.05), DHA-EE (*P <* 0.05) and DHA-PL (*P* < 0.05), while TC level in liver was significantly lower in mice fed diets with TG and EE compared with the control group (*P* < 0.05). At high fat level, mice fed diets with DHA-EE and DHA-PL had significantly lower hepatic TC level compared with the control diet (*P* < 0.05). Hepatic PL concentration experienced a significant increase in mice fed the diet with PL at high fat level (*P* < 0.05). Furthermore, both at low and high fat levels, hepatic DHA level significantly increased and AA level significantly decreased in all forms of DHA groups (*P* < 0.05), compared to control groups at two different fat levels, respectively. Additionally, cerebral DHA level in mice fed diets with DHA-FFA, DHA-EE and DHA-PL significantly increased compared with the control at high fat level (*P* < 0.05), but no significant differences were observed among dietary treatments for mice fed diets with low fat level.

**Conclusion:**

The present study suggested that not only total dietary fat content but also the molecular forms of omega-3 fatty acids contributed to lipid metabolism in mice. DHA-PL showed effective bioactivity in decreasing hepatic and serum TC, TG levels and increasing omega-3 concentration in liver and brain.

## Introduction

Omega-3 polyunsaturated fatty acids such as DHA and EPA contained in fish oil have long been suggested effective bioactive substances [1]. They have been proposed outstanding antithrombotic effects and efficient in prevention of cardiovascular diseases [2,3] through modulation of blood lipids [4] and lipoproteins [5]. The ingestion of long chain omega-3 polyunsaturated fatty acid (n-3 LC-PUFA) can also prevent or treat nonalcoholic fatty liver disease (NAFLD) [6-8]. Furthermore, omega-3 PUFA especially DHA have been recognized as key substances in signal transduction and as reservoirs of lipid messengers [4,9]. Based on the biological functions above, an increased intake of DHA and EPA has been recommended [10-12].

DHA and EPA in natural fish oil exist as triglycerides (TG) and phospholipids (PL), while they can also be ingested in the form of purified ethyl esters (EE) or free fatty acids (FFA) [13]. EE is the most common form in fish oil products, since it is convenient to concentrate highly-purified EE bound omega-3 PUFA. However, this formulation may lead to ethanol toxicity. TG bound omega-3 is a natural form of fish oil and is easily absorbed though purification may be a major problem. Effects of TG, EE and FFA forms of fish oil on lipid metabolism in mammals have been compared and remained to be controversial. Dyerberg reported that the bioavailability of FFA was not significantly different from natural triglycerides, whereas the bioavailability of EE was inferior to TG [14]. Most of other relevant research confirmed that TG is superior to EE [15-18]. However, Krokan suggested that bioavailability of EPA and DHA is as efficient in ethyl esters as in glyceryl esters [19]. Dietary phospholipids have been suggested biological and nutritional functions. As essential constituents of cell membrane, phospholipids, such as phosphatidylcholine (PC) and phosphatidylethanolamine (PE), are remarkable surface active agents and also believed to be good bioactive substances in resisting hypertriglyceridemia [20].

Recent research has suggested superior functions of PL bound DHA, including anti-inflammation [21], anti-oxidation [22], promoting the differentiation of erythroleukemia cancer cells [23,24] and antitumor functions [25]. Until recently few studies have focused on the comparison of effects between PL bound omega-3 and other formulations. Thus, the lack of a controlled study comparing those four particular forms of omega-3 fatty acid(TG, EE, FFA, PL) make it necessary to investigate the effects of different omega-3 fatty acid formulation on lipid metabolism in mice.

Researchers have suggested that not only chemical form but co-ingested fat level can contribute to bioavailability of omega-3 fish oil. Lawson and Hughes have reported that bioavailability of omega-3 s administered as standard ethyl ester form increased three-folds when ingested in a high fat meal, whereas triglycerides remain more stable in diets with different fat level [26].

Recently, Tanaka et al. [27] has reported the effect of long-term feeding and relatively high dosage of TG, EE, FFA and PL forms omega-3 PUFA on lipid absorption in mice. Up to date, however, the comprehensive effect of short-term, low dosage feeding of different omega-3 s formulations on lipid metabolism in mice, with different levels of fat context, hasn’t been reported. In the present study, we prepared TG, EE, FFA, PL forms of DHA and EPA and compared the effects of short term (1 week), low dosage (0.7%) of those four omega-3 formulations on lipid metabolism in mice. Additionally, the influence of different co-ingested fat level on lipid metabolism was also analyzed.

## Materials and methods

### Preparation of different forms of EPA and DHA

In the present study, four different forms of omega-3 PUFAs were prepared, including TG, EE, FFA and PL forms. Dietary DHA-PL was extracted from squid roe by the method of Folch [28], and purified by the silica gel chromatography. The proportion of PC and PE in dietary PL were 93.3% and 3.88%, respectively, with 2.82% for the remaining constituent, according to HPLC-ELSD analysis. The ratio of DHA to EPA in dietary PL was 2.6:1, determined by an Agilent 6890 Gas chromatography. To make 2.6:1 a standard for the ratio of DHA to EPA in each DHA supplement, two lipid products- a fish oil product (DHA%: 6.5%, EPA%: 22%) provided by Wanlian Pharmaceutical Inc., and Algamac microalgae powder (DHA%: 33.5%, EPA%: 0%) from Xiamen Huison Biotech Co., Ltd- were mixed as 1:1.5 to make dietary DHA-TG. DHA-FFA was a mixture of two oil products (X: DHA%: 65%, EPA%: 11%; Y: DHA%: 33%, EPA%: 25%) as X:Y = 1:1.14, purchased from Haiyuan Health Biological Science and Technology Co., Ltd (Hebei, China). TG bound omega-3 s (DHA-EE) was prepared by the method of esterification according to Li DW et al. [29], using DHA-FFA as substrate. The ratio of DHA to EPA in all prepared diets was approximately 2.6:1. The concentration of omega-3 PUFA for each omega-3 formulation was 8.5% for EPA and 22.4% for DHA in TG, 20.2% for EPA and 58.6% for DHA in EE, 20.3% for EPA and 56.6% for DHA in FFA, 8.9% for EPA and 23.3% for DHA in PL.

### Animals and diets

Male Balb/c mice (20 ± 2) were provided by Vital River Laboratories (Beijing, China),and randomly assigned to ten groups: Control group, TG group, FFA group, EE group and PL group in both low fat (LF) and high fat (HF) diet, with 6 mice in each group. The mice were housed individually in metal cages under a 12 h light/dark cycle, a constant temperature of 24 ± 1 °C, and a relative humidity of 65 ± 15% with free access to water and food for 7 days.

The basal diets were modified basing on AIN76, as shown in Table 1. To make omega-3 PUFA concentration equal in all the omega-3 diets, the supplement of fish oil in each group was adjusted basing on the omega-3 concentration in the different fish oil formulation prepared and the molar mass of PL, TG, EE or FFA. All omega-3 supplement groups contained 0.7% DHA + EPA, and the ratio of DHA/EPA is approximately 2.6 (Table 2).

**Table 1:** Formulation of the experimental diets (% dry matter)

Ingredient	LF-Control	LF-TG	LF-FFA	LF-PL	LF-EE	HF-Control	HF-TG	HF-FFA	HF-PL	HF-EE
Fish oil	0	23.75	9.05	30.35	9.51	0	23.75	9.05	30.35	9.51
Soybean oil	20	13.10	5.50	13.60	5.60	50	44.72	47.99	44.92	47.89
Lard oil	30	13.10	34.40	13.60	35.40	175	156.53	167.96	157.24	167.60
Cornstarch	150	150	150	150	150	75	75	75	75	75
Sucrose	500	500	500	500	500	400	400	400	400	400
Casein	200	200	200	200	200	200	200	200	200	200
Fiber	50	50	50	50	50	50	50	50	50	50
Mineral	35	35	35	35	35	35	35	35	35	35
Vitamin	10	10	10	10	10	10	10	10	10	10
Choline bitartrate	2	2	2	2	2	2	2	2	2	2
DL-meth	3	3	3	3	3	3	3	3	3	3

**Table 2:** Fatty acid composition of the experimental diets (g/kg)

Fatty acid	LF-Control	LF-TG	LF-FFA	LF-EE	LF-PL	HF-Control	HF-TG	HF-FFA	HF-EE	HF-PL
C14:0	0.45	1.68	0.51	0.53	0.70	2.62	3.82	2.51	2.51	2.85
C15:0	-	-	-	-	0.22	-	-	-	-	0.22
C16:0	9.14	13.21	8.48	8.72	11.35	45.54	49.45	43.75	43.65	47.60
C16:1	0.67	1.45	0.77	0.79	1.17	3.92	4.66	3.76	3.75	4.39
C17:0	-	0.59	-	-	-	-	0.59	-	-	-
C18:0	3.84	2.33	3.88	3.98	4.26	20.09	18.47	19.36	19.31	20.41
C18:1	17.08	9.73	16.35	16.76	9.77	85.87	78.17	82.78	82.57	78.25
C18:2	15.20	8.94	8.32	8.51	9.28	53.49	47.84	51.43	51.30	48.06
C18:3	1.42	0.93	0.39	0.40	0.97	3.55	3.17	3.41	3.40	3.19
C18:4	-	0.45	-	-	-	-	0.45	-	-	-
C20:1	-	-	0.23	0.21	2.19	-	-	0.23	0.21	2.19
C20:2	-	0.46	0.19	0.15	-	-	0.46	0.19	0.15	-
C20:4	-	-	0.25	0.24	0.55	-	-	0.25	0.24	0.55
C20:5	-	1.94	1.86	1.76	1.95	-	1.94	1.86	1.76	1.95
C22:1	-	-	0.36	0.31	-	-	-	0.36	0.31	-
C22:2	-	-	0.19	0.18	-	-	-	0.19	0.18	-
C22:4	-	0.94	0.26	0.26	0.33	-	0.94	0.26	0.26	0.33
C22:6	-	5.13	5.13	5.13	5.13	-	5.13	5.13	5.13	5.13
Total FA	47.80	47.76	47.18	47.93	47.87	215.07	215.09	215.47	214.72	215.10

Blood samples were collected by cardiac puncture after 12-hour fast. Livers, brains, kidneys, hearts, perirenal white adipose tissues and epididymal adipose tissues were excised and weighed immediately. Serum was extracted from blood sample by centrifuging the blood at 7500 × g for 15 min. All data were conducted according to the guidelines provided by the ethical committee of experimental animal care at Ocean University of China (Qingdao, China).

### Serum and hepatic lipids determination

The concentrations of serum TG and TC were determined with enzymatic reagent kits (Biosino, Beijing, China). Liver lipids were extracted with chloroform-methanol 2:1 as described by Folch et al. [28], and then dissolved with Triton X-100. The concentrations of hepatic TG and TC was measured with enzymatic reagent kits (Biosino, Beijing, China), and total phospholipids levels in liver were analyzed by the methods of Bartlett [30].

### Analysis of fatty acid composition

Lipids extracted from livers and brains were prepared by transmethylation with HCL/methanol and the fatty acid composition was analyzed with gas chromatograph.

An Agilent 6890 gas chromatograph, equipped with a flame-ionization detector, was used to analyze the composition of cerebral and hepatic fatty acid and also FA composition of diet in each group. The column was a HP-INNOWAX capillary column (30 m × 0.32 mm × 0.25 μm). The temperature of the detector and injector were kept constant at 250°C and 240°C, respectively, and the oven temperature was increased from 170°C to 240°C at 3°C/min and held at 240°C for 15 min. Nitrogen was used as carrier gas at the flow rate of 1.2 mL/min.

### Statistical analysis

The results are presented as mean ± standard deviations. All data were subjected to analysis of variance using the SPSS software (version 18.0; SPSS Inc., Chicago, IL, USA). Differences between the means were tested by one-way ANOVA, and all detected significant differences were further evaluated by Duncan multiple. The level of significance chosen was *P* < 0.05.

## Results

### General observation

After 7 days observation, there was no significant difference in daily food intake among 10 dietary treatments (*P* > 0.05). In low fat diet, body weight gain was significantly lower (*P* < 0.05) in LF-EE group (*P* < 0.05) and LF-PL group (*P* < 0.05), but not in LF-TG or LF-FFA group, compared with LF-control. Weight of epididymal adipose tissue of mice in LF-TG and LF-FFA was significantly lower (*P* < 0.05), compared to LF control group (*P* < 0.05). Body weight gain of mice in DHA-EE group was also significantly restrained (*P* < 0.05), and weight of perirenal adipose tissue in this group declined significantly (*P* < 0.05).

In high fat diet, heart weight of mice in PL group and weight of epididymal adipose tissue of FFA and EE group both increased significantly(*P* < 0.05), while no changes were examined in body weight of any group according to values of *P* < 0.05. (Table 3)

**Table 3:** Effects of different forms of n-3 FA on the growth of mice

Group	Body Weight Gain (g)	Liver Weight (g/100 g BW)	Kidney Weight (g/100 g BW)	Heart Weight (g/100 g BW)	Brain Weight (g/100 g BW)	Perirenal WAT (g/100 g BW)	Epididymal WAT (g/100 g BW)
LF-Control	1.58 ± 0.19^a^	5.09 ± 0.16^a^	1.69 ± 0.05^a^	0.49 ± 0.02^a^	1.45 ± 0.04^a^	0.43 ± 0.13^ab^	1.46 ± 0.13^ab^
LF-TG	1.32 ± 0.11^ab^	5.10 ± 0.04^a^	1.64 ± 0.02^a^	0.52 ± 0.01^a^	1.46 ± 0.03^a^	0.66 ± 0.07^a^	1.64 ± 0.06^a^
LF-FFA	1.20 ± 0.17^abc^	5.27 ± 0.16^a^	1.67 ± 0.03^a^	0.50 ± 0.01^a^	1.44 ± 0.05^a^	0.54 ± 0.04^a^	1.36 ± 0.08^abc^
LF-EE	0.70 ± 0.28^bc^	4.98 ± 0.07^a^	1.72 ± 0.05^a^	0.50 ± 0.01^a^	1.46 ± 0.05^a^	0.17 ± 0.09^b^	1.23 ± 0.12^bc^
LF-PL	0.50 ± 0.35^c^	4.99 ± 0.18^a^	1.67 ± 0.08^a^	0.49 ± 0.01^a^	1.46 ± 0.02^a^	0.24 ± 0.10^b^	1.00 ± 0.20^c^
HF-Control	2.28 ± 0.24^d^	4.90 ± 0.14^de^	1.71 ± 0.03^d^	0.51 ± 0.01^d^	1.44 ± 0.05^d^	0.30 ± 0.11^d^	1.19 ± 0.10^de^
HF-TG	2.22 ± 0.27^d^	4.60 ± 0.07^de^	1.71 ± 0.04^d^	0.51 ± 0.01^d^	1.53 ± 0.05^d^	0.16 ± 0.10^d^	1.00 ± 0.20^d^
HF-FFA	2.27 ± 0.10^d^	4.84 ± 0.06^de^	1.67 ± 0.06^d^	0.53 ± 0.02^de^	1.43 ± 0.02^d^	0.30 ± 0.09^d^	1.59 ± 0.16^f^
HF-EE	2.18 ± 0.13^d^	4.95 ± 0.17^d^	1.68 ± 0.04^d^	0.52 ± 0.03^d^	1.45 ± 0.05^d^	0.29 ± 0.07^d^	1.69 ± 0.08^f^
HF-PL	2.28 ± 0.20^d^	4.58 ± 0.06^e^	1.72 ± 0.03^d^	0.58 ± 0.03^e^	1.48 ± 0.04^d^	0.23 ± 0.05^d^	1.45 ± 0.05^ef^

WAT: Weight of adipose tissue BW: Body weight

a–c At low fat level, means in one column sharing the same superscript letter are not significantly different determined by Duncan Multiple (*P* > 0.05)

d–f At high fat level, means in one column sharing the same superscript letter are not significantly different determined by Duncan Multiple (*P* > 0.05)

### Serum lipids concentration

After one-week feeding, serum TC concentration in mice fed the diet with DHA-FFA, DHA-EE and DHA-PL at low fat level were significantly lower than that in the control group (*P* < 0.05), while for high fat level, no such difference was observed. Furthermore, there was no significant difference in serum TG concentration among dietary treatments with different forms of DHA ingestion at either low fat or high fat level. (Table 4)

**Table 4:** Effects of different forms of n-3 FA on serum lipids concentration

Group	TG	TC
LF Control	180 ± 20^ab^	210 ± 8^a^
LF-TG	215 ± 15^a^	194 ± 4^ab^
LF-FFA	213 ± 9^a^	173 ± 4^bc^
LF-EE	139 ± 19^b^	126 ± 12^d^
LF-PL	150 ± 18^b^	145 ± 9^cd^
HF Control	137 ± 18^ef^	157 ± 17^e^
HF-TG	138 ± 22^ef^	145 ± 10^e^
HF-FFA	189 ± 21^e^	175 ± 11^e^
HF-EE	129 ± 15^f^	172 ± 15^e^
HF-PL	159 ± 13b^f^	161 ± 9^e^

a–d At low fat level, means in one column sharing the same superscript letter are not significantly different determined by Duncan Multiple (*P* > 0.05)

e–f At high fat level, means in one column sharing the same superscript letter are not significantly different determined by Duncan Multiple (*P* > 0.05)

### Hepatic lipids concentrations

In short-term feeding of omega-3 s, hepatic TG level decreased significantly in DHA-TG (*P* < 0.05), DHA-EE (*P* < 0.05) and DHA-PL (*P* < 0.05) groups, while hepatic TC level decreased significantly in DHA-TG and DHA-EE groups (*P* < 0.05), in low fat diets. While in high fat groups, hepatic TC level decreased significantly in DHA-EE and DHA-PL group in high fat context(*P* < 0.05). Hepatic PL level experienced a significant increase in PL group in high fat diet (*P* < 0.05). (Table 5)

**Table 5:** Effects of different forms of n-3 FA on hepatic lipids concentration

Group	TG	TC	PL
LF Control	47.6 ± 10.2^a^	2.74 ± 0.22^a^	33.0 ± 1.3^a^
LF-TG	26.3 ± 2.8^b^	2.22 ± 0.13^b^	29.5 ± 1.5^a^
LF-FFA	31.3 ± 3.0^ab^	2.52 ± 0.07^ab^	31.2 ± 0.9^a^
LF-EE	16.9 ± 4.1^b^	2.17 ± 0.23^b^	31.9 ± 1.7^a^
LF-PL	22.3 ± 5.7^b^	2.58 ± 0.08^ab^	33.2 ± 0.7^a^
HF Control	38.0 ± 2.1^c^	2.55 ± 0.12^c^	30.6 ± 0.4^c^
HF-TG	36.2 ± 4.8^c^	2.45 ± 0.16^cd^	32.9 ± 1.3^cd^
HF-FFA	46.5 ± 7.8^c^	2.56 ± 0.13^c^	32.3 ± 0.7^cd^
HF-EE	35.3 ± 7.3^c^	1.97 ± 0.28^d^	33.3 ± 1.5^cd^
HF-PL	34.3 ± 2.5^c^	1.97 ± 0.11^d^	34.5 ± 0.8^d^

a–b At low fat level, means in one column sharing the same superscript letter are not significantly different determined by Duncan Multiple (*P* > 0.05)

c–d At low fat level, means in one column sharing the same superscript letter are not significantly different determined by Duncan Multiple (*P* > 0.05)

### Fatty acid composition of liver

Hepatic fatty acid composition, especially the level of DHA and AA, changed drastically after one-week feeding of omega-3 s. For mice fed diets with low fat level, hepatic DHA level in mice fed diets with DHA-TG, DHA-FFA, DHA-EE and DHA-PL were significantly increased by 49.4%, 32.9%, 38.8% and 61.2% (*P* < 0.05) respectively compared with the LF-control group. For mice fed diets with high fat level, hepatic DHA level in mice fed diets with DHA-TG, DHA-FFA, DHA-EE and DHA-PL were also significantly increased by 50.6%, 44.3%, 36.7%, 53.1% (*P* < 0.05) separately compared to the HF-control. However, at both fat level, AA level was significantly decreased in all forms of DHA groups (*P* < 0.05). (Table 6)

**Table 6:** Effects of different forms of n-3 FA on main hepatic FA composition

Group	C16:0	C16:1n-6	C18:0	C18:1n-9	C18:2n-6	C20:4n-6	C22:6n-3
LF-Control	21.8 ± 0.3^a^	1.75 ± 0.16^ab^	13.3 ± 1.0^a^	20.1 ± 1.9 ^a^	12.5 ± 2.0^a^	11.7 ± 1.3^a^	8.5 ± 0.3^a^
LF-TG	24.0 ± 0.2^b^	2.08 ± 0.17^b^	13.5 ± 0.3^a^	18.5 ± 1.3^ab^	9.0 ± 0.2^b^	7.72 ± 0.12^b^	12.7 ± 0.4^bd^
LF-FFA	24.0 ± 0.2^b^	1.77 ± 0.21^ab^	14.1 ± 0.8^a^	19.6 ± 1.6^ab^	10.1 ± 0.4^ab^	7.27 ± 0.20^b^	11.3 ± 0.4^c^
LF-EE	23.2 ± 1.0^ab^	1.40 ± 0.21^a^	14.0 ± 1.2^a^	15.8 ± 1.3^bc^	12.1 ± 0.5^a^	8.71 ± 0.56^b^	11.8 ± 0.4^bc^
LF-PL	25.1 ± 0.4^b^	1.66 ± 0.29^ab^	14.6 ± 1.1^a^	13.9 ± 1.1^c^	12.9 ± 0.5^a^	8.06 ± 0.24^b^	13.7 ± 0.2^d^
HF-Control	22.6 ± 0.4^e^	0.81 ± 0.07^e^	13.7 ± 0.5^e^	15.7 ± 1.1^e^	16.0 ± 1.4^e^	12.2 ± 0.7^e^	7.9 ± 0.2 ^e^
HF-TG	23.2 ± 0.8^ef^	0.74 ± 0.19^e^	13.7 ± 1.1^e^	13.8 ± 1.3^e^	16.3 ± 1.1^e^	8.14 ± 0.52^f^	11.9 ± 0.4^f^
HF-FFA	24.0 ± 0.9^ef^	0.86 ± 0.21^e^	13.7 ± 1.1^e^	14.9 ± 1.7^e^	16.1 ± 0.9^e^	8.31 ± 0.43^f^	11.4 ± 0.7^f^
HF-EE	24.6 ± 0.4^f^	0.97 ± 0.07^e^	11.9 ± 0.2^e^	16.4 ± 0.8^e^	17.5 ± 0.5^e^	8.05 ± 0.19^f^	10.8 ± 0.5^f^
HF-PL	24.7 ± 0.8^f^	0.95 ± 0.08^e^	13.7 ± 0.9^e^	14.6 ± 0.9^e^	17.5 ± 0.9^e^	7.78 ± 0.52^f^	12.1 ± 0.2^f^

a–d At low fat level, means in one column sharing the same superscript letter are not significantly different determined by Duncan Multiple (*P* > 0.05)

e–f At high fat level, means in one column sharing the same superscript letter are not significantly different determined by Duncan Multiple (*P* > 0.05)

### Fatty acid composition of brain

Cerebral DHA level of DHA-FFA, DHA-EE and DHA-PL groups experienced a significant increase in high fat diets (*P* < 0.05), by 9.1%, 9.1% and 11.0% respectively, compared with the high fat control group. There was no significant change of cerebral DHA level in HF-TG group, but with an increase of 5.5% compared with HF-control group. No significant changes of DHA level were observed for low fat diets. (Table 7)

**Table 7:** Effects of different forms of n-3 fatty acid on main cerebral FA composition in mice

Group	C14:0	C16:0	C18:0	C18:1n-9	C18:2n-6	C20:4n-6	C22:6n-3
LF-Control	3.2 ± 0.8^ab^	25.0 ± 3.6^a^	27.9 ± 4.1^ab^	19.9 ± 1.1^ab^	0.53 ± 0.22^a^	8.2 ± 1.8^a^	15.2 ± 2.5^a^
LF-TG	2.6±0.6^b^	23.4±3.6^a^	30.1±3.2^a^	18.5±0.4^b^	0.52±0.08^a^	8.4±1.2^a^	16.5±2.8^a^
LF-FFA	3.4±0.5^a^	26.9±2.5^a^	25.5±1.3^b^	20.6±1.4^a^	0.63±0.15^a^	8.3±0.9^a^	14.6±2.1^a^
LF-EE	2.8 ± 0.3^ab^	25.8 ± 2.0^a^	26.0 ± 0.6^ab^	18.8 ± 1.8^b^	0.6 ± 0.09^a^	9.0 ± 0.5^a^	14.9 ± 2.2^a^
LF-PL	2.8±0.4^ab^	25.0±3.4^a^	28.2±3.5^ab^	19.0±1.2^b^	0.6±0.07^a^	8.6±0.8^a^	15.8±1.9^a^
HF-Control	2.5±0.5^c^	25.4±0.9^c^	25.9±0.9^c^	18.8±0.6^c^	0.74±0.02^c^	10.2±1.2^c^	16.4±1.1^c^
HF-TG	2.7 ± 0.3^c^	23.7 ± 3.0^c^	29.2 ± 5.4^c^	17.6 ± 1.7^c^	0.81 ± 0.12^cd^	8.8 ± 0.8^d^	17.3 ± 0.5^cd^
HF-FFA	2.6 ± 0.3^c^	23.0 ± 3.0^c^	28.3 ± 3.3^c^	17.8 ± 1.1^c^	0.94 ± 0.28^d^	9.4 ± 0.7^cd^	17.9 ± 1.2^d^
HF-EE	2.5±0.7^c^	23.9±2.3^c^	26.7±2.6^c^	18.3±0.6^c^	0.78±0.08^cd^	9.9±0.2^cd^	17.9±1.1^d^
HF-PL	2.3±0.5^c^	22.7±2.6^c^	27.5±2.8^c^	18.0±0.6^c^	0.77±0.11^cd^	10.4±1.2^c^	18.2±1.5^d^

a–b At low fat level, means in one column sharing the same superscript letter are not significantly different determined by Duncan Multiple (*P* > 0.05)

c–d At high fat level, means in one column sharing the same superscript letter are not significantly different determined by Duncan Multiple (*P* > 0.05)

## Discussion

In order to compare the effects of TG, EE, FFA and PL forms of omega-3 s on lipid metabolism in mice, the ratio of DHA to EPA and their respective amount in diets were confirmed to be statistically equal in each DHA supplement group. Furthermore, fat level in diets was considered, for previous study has suggested that fat level in diets can significantly affect bioavailability of omega-3 fish oil [26].

Following 1 week feeding of omega-3 s, serum TC and TG levels in mice fed diets with DHA-PL, DHA-FFA and DHA-EE were significantly decreased at low fat compared to LF control, while no significant difference were observed among high fat fed mice (Table 4). The result suggested that PL, FFA and EE forms of omega-3 s were effective in inhibiting serum lipids concentration compared with TG. Additionally, the results implied that high fat content in diets may restrain the therapeutic effect of omega-3 s on hyper-triglyceridemia compared with low fat content. This result was different from Lawson et al., who reported that high fat content could increase the bioavailability of DHA, other than low fat content [26]. The difference implied that high fat content was effective in increasing the bioavailability of DHA but not the efficiency of DHA in lowering TC and TG concentrations in serum. The inefficiency of omega-3 supplement in high fat fed mice was probably based on its lower ratio in the total fat content, for the ratio of DHA + EPA in diet was 0.7% in both high fat and low fat groups, while the ratio of total fat content in diet increased a lot in high fat groups.

At low fat level, the decrease of TC and TG level in liver mainly existed in PL, TG and EE groups, and no significant difference was observed between TG and EE. Especially, hepatic PL concentration experienced a significant increase in PL group for high fat diet. These results suggested better effect of PL, TG and EE bound DHA in decreasing hepatic TC, TG levels compared with FFA, and equal efficiency of EE and TG in decreasing TC and TG level in liver. This was different from the previous study [31], in which DHA-TG reduced liver TG level more effectively than DHA-EE after one-week feeding. The indifference between EE and TG forms in the present study was probably due to the lower dosage of DHA + EPA (0.7%) supplement. PL bound DHA is efficient in increasing PL level in liver. This could probably account for the increase of hepatic DHA level, because DHA-PL is a major form for DHA in liver.

Tanaka [27] fed mice with TG, EE, FFA and PL forms of DHA supplement (1.5%) in the low-fat diet (6%) for 4 weeks to assess the effects of molecular form on distribution of omega-3 into organs. In the present study, short-term effect of low dosage of DHA + EPA (0.7%) on fatty acid composition in liver and brain of mice were investigated in high fat or low fat content. The ingestion of all the four forms of omega-3 s significantly increased hepatic DHA content. Furthermore, we found total fat content in diets a key factor in influencing the effect of different forms of omega-3 s on hepatic DHA concentration. When low dosage of DHA was fed to mice in low fat context, the effect of DHA-PL was more effective than TG, EE and FFA forms in increasing hepatic DHA contents.

Recent studies have shown possible therapeutic and preventive effects of cerebral DHA on many psychiatric diseases such as attention deficit hyperactivity disorder [32,33], depression [34], Alzheimer’s [35], and, probably Parkinson’s disease [36]. In the present study, cerebral DHA concentration of mice fed diets with low fat was found to have no significant change after 1 week feeding. However, cerebral DHA concentration increased significantly in mice fed diets with FFA, EE and PL at high fat by 9.1%, 9.1% and 11.0% respectively. There was no significant increase for cerebral DHA level in HF-TG group but with a 5.5% higher level compared to HF-control group. Results of this study suggested that in short-term feeding of omega-3 s, the increase of DHA level is much more significant in liver than in brain, and also, indicated that the efficiency of dietary DHA supplement on cerebral DHA content would be affected dramatically by co-ingested fat content, which implied a dietary suggestion when consuming omega-3 s. A series of behavioral experiment [37-40] have shown that DHA intake could particularly increase cerebral DHA level, and as a consequence, improve learning and memory abilities of rats or mice. Most of those studies are carried out with a long period feeding of DHA. Our present study indicated that short-term feeding of omega-3 in high fat meals could also increase cerebral DHA level.

The present results indicated that DHA-PL could increase DHA concentration in liver and brain during a short period. The efficiency of PL bound omega-3 s is more significant than EE or FFA forms in increasing DHA concentration in tissues. This result was probably due to the difference in absorption and distribution of different omega-3 formulation. When dietary lipids are consumed, they are digested mainly in intestine, where the lipids are broken into free fatty acids and other remaining substances, such as monoglycerides, ethanol or lyso-phosphatidylcholine and they are absorbed by enterocytes of the intestinal wall. Afterwards those absorbed FFAs and other substances are reassembled into triglyceride and phospholipids. For EE form fatty acids, this is obviously a less efficient process than direct absorption of triglycerides [14], which may lead to a worse bioavailability of EE form omega-3 s compared with TG form. In the present study, the superior bioavailability of PL bound DHA was probably due to its amphiphilic character, which might influence the surface composition of fat droplets thus facilitate the binding of hydrolyzing enzymes and hence the digestion [41]. Otherwise, PL was one of the main forms for DHA in organs, especially in brains, for which the ingestion of DHA-PL might increase the efficiency in short-term incorporation of DHA into organs.

Up to date, TG and EE are two main forms of fish oil in the market. TG is the natural form of fish oil, while EE form of omega-3 is the most common form in industrial fish oil products because of convenience in synthesis and purification. However, results of the present study suggested that DHA-PL was also a bioactive form. Furthermore, as a natural form of fish oil, PL was much safer compared with EE or FFA form. Additionally, it has been reported in a 10-week oxidation study that the stability of DHA-EE decayed 33% more rapidly than DHA-PL [42]. Thus, PL was suggested a better form for dietary omega-3 fatty acid.

## Conclusion

The present study suggested that not only total dietary fat content but the forms of omega-3 fatty acids contributed to the effects of Omega-3 FA on lipid metabolism in mice. DHA-PL was effective in decreasing hepatic and serum TC, TG levels and increasing omega-3 concentration in liver and brain.

## List of Abbreviations

DHA: Docosahexaenoic acid; EPA: Eicosapentaenoic acid; TG: Triglyceride; EE: Ethyl ester; FFA: Free fatty acid; PL: Phospholipid; FA: Fatty acid; TC: Total cholesterol; WAT: Weight of adipose tissue; BW: Body weight.

## Misc

Xiao Tang and Zhao-Jie Li contributed equally to this work

## Competing interests

The authors declare that they have no competing interests.

## Authors’ contributions

XT and ZJL substantially contributed to the design of the study, performing the experiment, assembly, analysis of data, and drafting the manuscript. JX and YX participated in experimental work and collection, assembly. JZL contributed to performing the experiment. JFW and TY participated in designing and funding the study, CHX and YMW made contributions to design, analysis and revision of the manuscript, as well as funding the study. All authors have read and approved this manuscript.

## Author’s information

^1^College of Food Science and Engineering, Ocean University of China, Qingdao, China

^2^ Department of Applied Biological Sciences, Saga University, Saga, Japan
